# Low-Power AlGaN/GaN Triangular Microcantilever for Air Flow Detection

**DOI:** 10.3390/s23177465

**Published:** 2023-08-28

**Authors:** Balaadithya Uppalapati, Durga Gajula, Manav Bava, Lavanya Muthusamy, Goutam Koley

**Affiliations:** 1 Holcombe Department of Electrical and Computer Engineering, Clemson University, Clemson, SC 29634, USA; 2 School of Electrical and Computer Engineering, Georgia Institute of Technology, Atlanta, GA 30332, USA; 3 Department of Physics and Astronomy, Clemson University, Clemson, SC 29634, USA

**Keywords:** MEMS, AlGaN/GaN, two-dimensional electron gas, microcantilever heater, airflow detection

## Abstract

This paper investigates an AlGaN/GaN triangular microcantilever with a heated apex for airflow detection utilizing a very simple two-terminal sensor configuration. Thermal microscope images were used to verify that the apex region of the microcantilever reached significantly higher temperatures than other parts under applied voltage bias. The sensor response was found to vary linearly with airflow rate when tested over a range of airflow varying from 16 to 2000 sccm. The noise-limited flow volume measurement yielded ~4 sccm resolution, while the velocity resolution was found to be 0.241 cm/s, which is one of the best reported so far for thermal sensors. The sensor was able to operate at a very low power consumption level of ~5 mW, which is one of the lowest reported for these types of sensors. The intrinsic response time of the sensor was estimated to be on the order of a few ms, limited by its thermal properties. Overall, the microcantilever sensor, with its simple geometry and measurement configurations, was found to exhibit attractive performance metrics useful for various sensing applications.

## 1. Introduction

In recent years, there has been significant progress in developing sensors and actuators using microelectromechanical systems (MEMS) sensing elements, which have enabled the realization of highly sensitive, low-power, and miniaturized sensors [1,2]. There are widespread applications of these sensors, including the detection of mass attachment as well as changes in pressure, stress, force, and temperature [3,4,5]. Important applications in chemical and biological sensing, including gases, vapors, ions, and various bio-analytes [6,7,8,9,10], are also possible. MEMS sensors have also been utilized for performing air flow measurements that are very important for a large variety of industrial and medical applications [11,12]. MEMS flow sensors are typically based on a heated filament and are called thermal flow sensors, which measure the fluid flow sensor by detecting changes in temperature caused by the flow of the fluid. They are often used in harsh environments, such as in the automotive, aerospace, and chemical industries, due to their ease of fabrication, high sensitivity, fast response times, and lack of moving parts [13,14]. Several types of thermal flow sensors exist, including those made with silicon-on-insulator (SOI) and complementary metal-oxide-semiconductor (CMOS) technology [15,16]. Depending on the targeted working temperature, these sensors can be made using various heating and sensing materials, such as metals, semiconductors, polymers, and ceramics. The most commonly used heating elements are silicon oxide/nitride membranes and metallic heaters; however, metal-based heaters can suffer from reliability issues due to the electromigration of metal atoms [17]. To achieve uniform temperature distribution, complex geometries are necessary for metal-based heaters [18]. To address these issues, heated structures fabricated using doped semiconductor materials have been developed recently [18,19].

Although silicon-based sensors have dominated heated sensor applications so far, they are limited in their ability to operate at high temperatures above a few hundred °C due to degradation in the electronic properties of Si at higher temperatures [20,21]. To address this limitation, other materials such as III-Nitrides and silicon carbide (SiC) have been used for harsh environmental applications [22,23,24]. These materials have a wide bandgap, good thermal conductivity, and are chemically inert, making them well-suited for harsh environmental operations [25]. SiC-based micro-hotplates have recently been demonstrated for chemical sensing at elevated temperatures. Due to the difficulty of etching SiC-based suspended MEMS structures, this is harder to achieve. Suspended structures are required to reduce thermal mass and achieve fast response times and low power consumption. Additionally, complicated fabrication steps make integration with external devices challenging [26]. In contrast, the AlGaN/GaN microcantilevers utilized in this work use commercially available AlGaN/GaN-on-Si wafers and a standard Si release process (Bosch process), allowing for seamless integration with GaN as well as Si electronics. GaN-based microsystems are ideal for sensing applications in harsh environments such as combustion exhaust pipes, industrial processes, and oil and gas wells due to GaN’s inherent tolerance to temperature and chemicals. On the other hand, mature Si technology enables the realization of processing circuitry integrated on the same chip. AlGaN/GaN heterostructures have already been commercialized for high-frequency and high-power electronics because of the highly conductive two-dimensional electron gas (2DEG) at the AlGaN/GaN interface [27], and these sensors can leverage the already mature materials and device technology.

In this paper, we have investigated a triangular microcantilever-based flow sensor that utilizes a two-dimensional electron gas (2DEG) formed at the AlGaN/GaN interface (due to the piezoelectric properties of these materials) [3,4,5,6] as the conducting channel to heat the cantilever apex to perform airflow detection. A linear sensor response was observed over a wide range of airflow from 16 to 2000 sccm, which yielded a noise-limited airflow measurement resolution of ~4 sccm and a velocity resolution of 0.241 cm/s, which is one of the best values reported so far. Combined with their low power consumption of ~5 mW, these sensors offer attractive application potential in various applications, including wearable and remote flow sensing.

## 2. Materials and Methods

**Figure 1:**
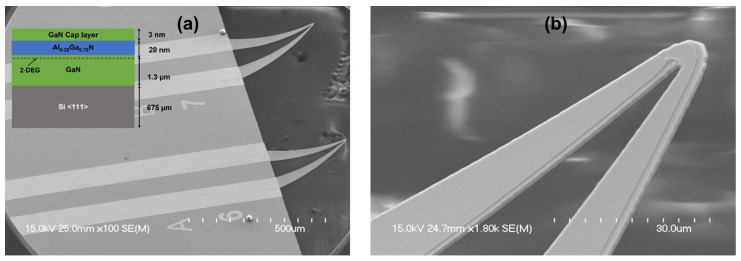
(**a**) SEM image of two AlGaN/GaN single-channel triangular microcantilevers with heights of 330 µm (left) and 300 µm (right), used for airflow detection. The inset shows the layered structure of the epitaxial wafer used for fabricating the sensors; (**b**) Magnified SEM image of the cantilever shows the details of the microcantilever apex.

The single-channel microcantilevers were fabricated using epitaxially grown AlGaN and GaN layers on Si (111) substrates purchased from NTT, Japan. The epitaxial layers consist of a buffer layer, an active layer of GaN (1 µm), and an AlGaN layer (20 nm), forming a 2DEG due to the polarization gradient at the AlGaN/GaN interface. A GaN cap layer (3 nm) was added to prevent the oxidation of AlGaN. A schematic of the layer structures is shown in the inset of Figure 1b. The microcantilever sensors were fabricated by following well-established procedures established earlier [28,29]. In the first step, the AlGaN barrier layer was etched to a depth of 100 nm using BCl_3_/Cl_2_ plasma chemistry, leaving the channel mesa areas with 2DEG formed at the AlGaN/GaN heterostructure interface. After the channel mesa formation, a metal stack of Ti (20 nm)/Al (100 nm)/Ti (45 nm)/Au (55 nm) was deposited at the base of the microcantilever, followed by rapid thermal annealing at 825 °C for 1 min to form the ohmic contacts. Finally, metal contact pad layers of Ti (20 nm)/Au (250 nm) were deposited for wire bonding and external probing. In the final step, to release the microcantilevers, a Bosch process was performed for through-wafer etching of the Si substrate from the backside, with patterned plasma-enhanced chemical vapor-deposited SiO_2_ used as a hard mask. The fabrication process steps are shown in Figure S1.

**Figure 2:**
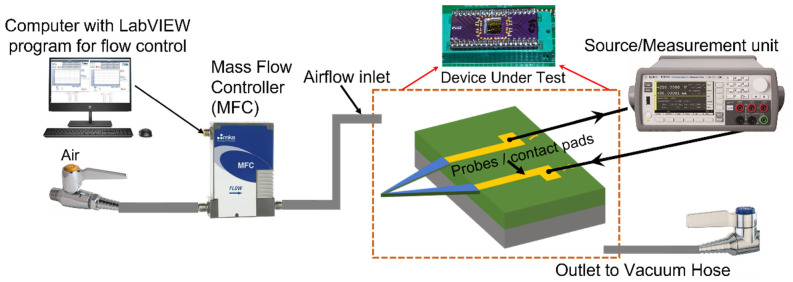
Experimental setup to investigate the airflow detection capability of the III-Nitride triangular microcantilever sensors. Inset shows a picture of the sensor chip wire-bonded to a custom-designed chip carrier.

High-resolution scanning electron microscope (HRSEM) images were taken (with Hitachi S-4700 FE-SEM (Hitachi, Ltd., Tokyo, Japan) at 15 kV bias) to reveal the structure of fabricated microcantilevers. Figure 1a shows the SEM image of two typical triangular microcantilevers, while Figure 1b is a magnified image around the cantilever apex, offering a side view and finer details of the AlGaN and GaN epitaxial layers. The nominal length of the arms of the cantilevers in Figure 1a was ~330 µm (left) and 300 µm (right), which tapers from a base width of 65 µm to 2 µm at the apex. These variations are due to fluctuations in lithographic patterning. In our experiments, we used microcantilevers with dimensions of 65 µm base width, 300 µm arm length (to be referred to henceforth as sensor 1), and 110 µm base width, 330 µm arm length (to be referred to as sensor 2). Results from two different microcantilevers are presented to establish the general validity of the detection mechanism as well as investigate the difference in response due to these variations. The experimental setup for the characterization of the microcantilever sensor included a mass flow controller (MFC) operated through a LabVIEW program, which automatically controlled the airflow rate into the sensor test chamber, as shown in Figure 2. The inlet, outlet, and external wiring ports of the test chamber were designed together, and their entire integrated assembly was 3D printed. The outlet port of the test chamber was connected to a vacuum hose to draw in the air, with flow regulated by the MFC. Due to the range limitations of the MFC used, the minimum and maximum airflow that could be set were 16 and 2000 sccm, respectively. For the lower range of airflow, i.e., from 16 sccm to 100 sccm, a 0.053 cm fitting was used at the inlet port for flowing the air on the microcantilever device under test (DUT). On the other hand, for airflow between 100 sccm and 2000 sccm, a 0.635 cm inlet port was used to flow the air to the DUT. The distance between the inlet tube and the sensor was maintained at 15 and 5 mm for the higher and lower flow measurement ranges, respectively. Two different diameters and distances were used for the inlet tubes to ensure sufficient air velocity near the microcantilever, which is needed for the efficient removal of heat from the heated cantilever apex. Essentially, this method, along with adjusting the distance between the nozzle end and the microcantilever (see discussion later), can be used to adjust the range of airflow that the microcantilever can detect as well as tune its flow detection sensitivity.

For airflow testing and characterization, the sensor chip was attached to a custom-designed printed circuit board (PCB) chip carrier, and the metal contact pads were wire-bonded to external leads of the PCB (see Figure 2 inset), which were biased using a source measurement unit (SMU, Keysight B2902 A). The microcantilever sensor was biased at a constant dc voltage of 10 V during measurements to maintain a steady-state temperature at the apex of the microcantilever. The current responses at different flow rates were recorded using the SMU. The thermal profile of the microcantilever at different bias voltages was recorded by an Inframetrics SC1000 Thermacam FLIR forward-looking infrared video camera, which indicated variation in the temperature profile under various biasing conditions.

## 3. Results and Discussion

**Figure 3:**
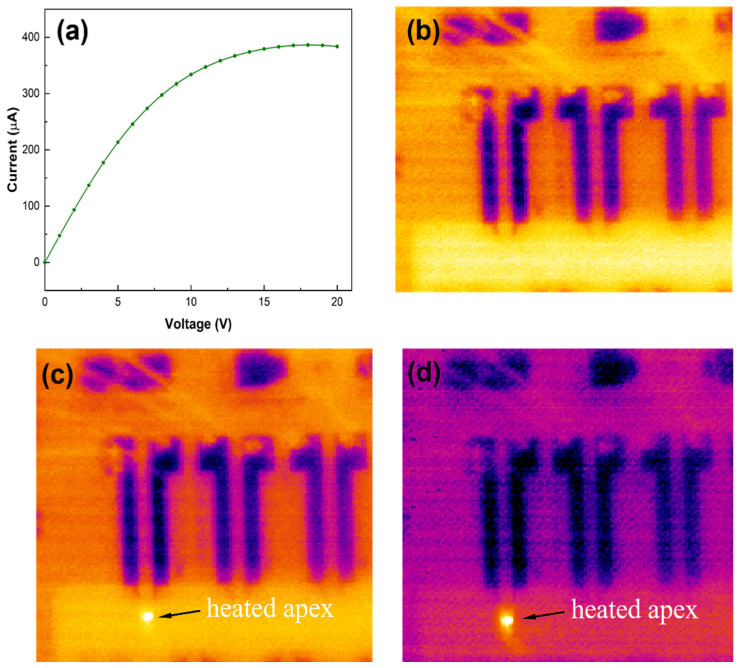
(**a**) *I–V* characteristics for the triangular microcantilever sensor 1. The current is found to saturate beyond 10 V. Thermal camera images for different voltage biases: (**b**) 0 V, (**c**) 10 V, and (**d**) 20 V. Heated apex of the microcantilever is pointed out by arrows in (**c**,**d**).

The current–voltage (*I*–*V*) characteristics of the microcantilever sensor are shown in Figure 3a. We observe that the current saturates for voltage biases >10 V, which is commonly observed for AlGaN/GaN high electron mobility transistors at zero gate bias and can be attributed to self-heating and 2DEG depletion [30,31]. To confirm that the apex of the tip is heating up under applied bias, we performed infrared (IR) microscope imaging under various biasing conditions. Figure 3b shows a thermal image of the microcantilever without any applied bias, while Figure 3c,d show thermal images of the heated apex region of the microcantilever under applied biases of 10 and 20 V, respectively. With no applied bias, we do not see any contrast between the apex of the cantilever and other parts, while under the applied bias of 10 V, significant contrast is observed between the apex and other parts of the cantilever. This contrast increases further under a 20 V bias, clearly indicating that the temperature of the apex increases as the bias is increased from 0 V, as expected. Our previous results indicate that the temperature can reach as high as 105 °C under a 20 V applied bias [32].

**Figure 4:**
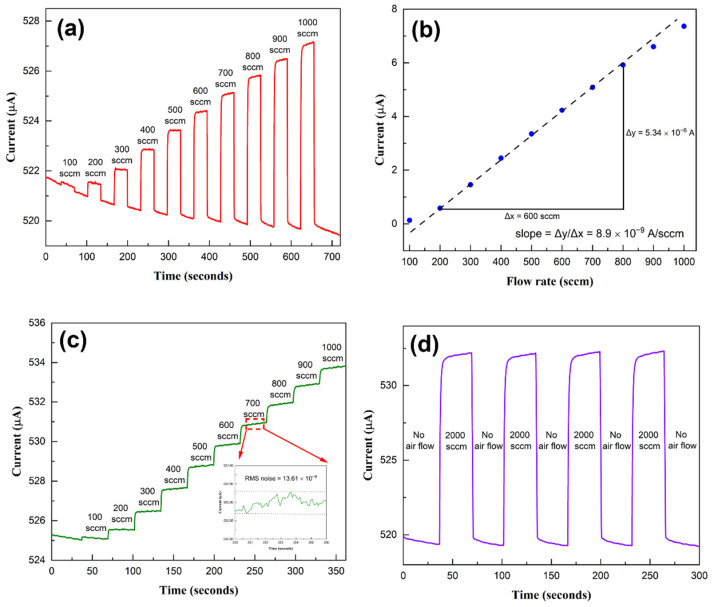
(**a**) Temporal response of the microcantilever sensor 2 under various flow rates over the range of 100 sccm to 1000 sccm. The flow was paused, i.e., set to 0 sccm, after each test flow rate. (**b**) Current change response corresponding to each flow rate plotted vs. flow rate, which shows a good linear correlation, (**c**) Sensor response with airflow varying stepwise from 100 to 1000 sccm at 100 sccm increments without any intermittent flow pause (inset shows the rms noise for the 700 sccm step flow), and (**d**) sensor response to 4 on/off cycles of 2000 sccm air flow. For all the flow measurements, the sensor voltage bias was maintained at 10 V.

To investigate the capability of the microcantilever sensor in sensing air flow, it was initially exposed to airflow rates varying from 100 to 1000 sccm, with a step size of 100 sccm and a sequential pause (flow rate reduced to 0) after each flow test level. The sensor’s responses to these airflow rates are shown in Figure 4a. We observe an envelope to the sensor response, which is likely caused by the charge instability typically observed in III-Nitrides [33,34]. Since this is contributed to a large extent by the bare surface, passivating the surface using a coating of SiN_x_ can be very useful in reducing such transients [35,36]. The sensor response, i.e., change in current magnitude, corresponding to each flow rate, has been plotted against the flow rate in Figure 4b. We find that the current change varies linearly with the airflow, increasing at a rate of ~8.9 nA/sccm. The sensor response was also recorded when the airflow rate was varied continuously from 100 to 1000 sccm without an alternate reset to zero flow rate, and the responses are shown in Figure 4c. The inset of Figure 4c shows the RMS noise for the 700 sccm step. To test the repeatability of the sensing response, multiple cycles of 2000 sccm flow rates, followed by a reset to 0 sccm, were recorded, and the responses are shown in Figure 4d. We find the sensor responses for the two to be practically identical, which highlights their stability and repeatability. The response of another sensor with airflow varying from 300 sccm to 1000 sccm is shown in Figure S2a, with flow pauses after each flow level. The response was also recorded with the airflow rate varied as a step function from 300 to 1000 sccm, without any reset to 0 sccm, as shown in Figure S2b. Figure S2c shows repeatable sensor response at multiple 2000 sccm flow cycles with airflow paused in between. Once again, excellent stability in responses can be observed.

We would like to mention here that the main principle of operation of the sensor is a reduction in temperature at the heated cantilever apex as the heat from that region is conducted away by the air flow. As the temperature of the apex is reduced, the resistance is reduced, which causes the current to increase, as seen from the sensing responses. Detailed analysis and modeling of the reduction in temperature can be performed using a similar approach to the model development for a heated cantilever apex for volatile organic compound (VOC) detection published earlier [37].

**Figure 5:**
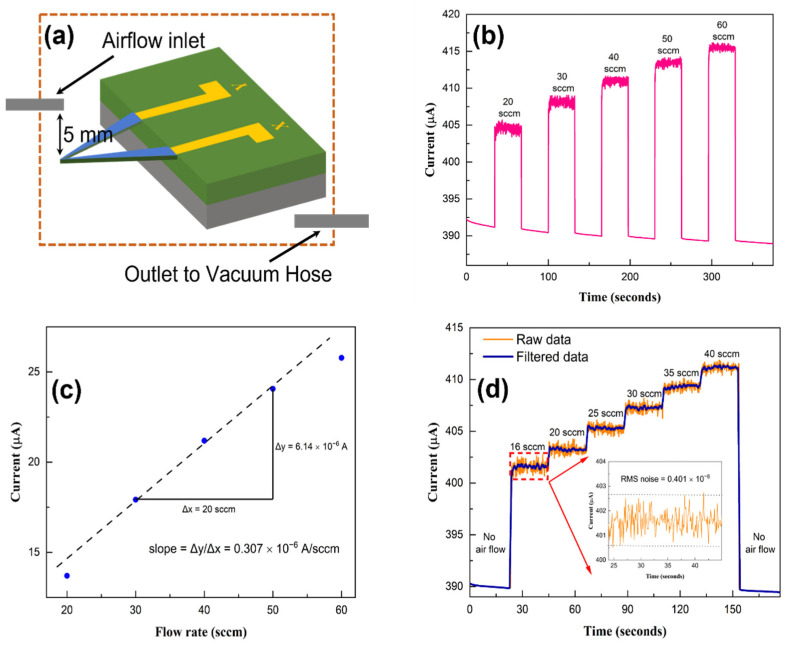
(**a**) Schematic of a modified measurement set-up with a narrower tube and a smaller gap (5 mm) between the airflow tube edge and the sensor 1; (**b**) Sensor response for air flow varying from 20 to 60 sccm; (**c**) Current change for various flow rates plotted against the flow rate; (**d**) Small incremental change in airflow from 16 to 40 sccm with a step size of 5 sccm (4 sccm for the first step). The inset shows a magnified plot of the first flow rate (16 sccm) step, where the random fluctuations in current are used for determining the RMS current noise, which turns out to be 0.401 μA.

The sensor response was also tested for lower flow rates in the range of tens of sccm. For this, a reduced-diameter (0.53 mm) flow tube was used, with closer positioning of the sensor from the tube end, to enhance the velocity of the air reaching the cantilever sensor. This helped to increase the rate of heat loss from the cantilever apex, enhancing the flow-sensing response of the microcantilever. It should be noted that a larger diameter tube (6.35 mm) was used for the higher flow rate sensing since a high flow rate with a narrow diameter tube would result in large mechanical stress and potential damage to the microcantilever sensor. Nonetheless, this method of adjusting the tube diameter and distance can be very useful in tuning the dynamic range and sensitivity of the sensor. Figure 5a shows a schematic diagram showing the narrower air flow tube in close proximity (5 mm away) to the microcantilever to enhance its detection sensitivity. The microcantilever sensor was exposed to varying airflow rates, from 20 to 60 sccm, in increments of 10 sccm, and the responses are shown in Figure 5b. The current change responses of the sensor as a function of the airflow rates are shown in Figure 5c. Figure 5d shows the sensor response for airflow rates varying from 16 sccm to 40 sccm at even smaller increments of 5 sccm. The inset of Figure 5d shows a magnified plot of the first flow rate (16 sccm) step, where the random fluctuations in current (due to a combination of air flow turbulence as well as sensor temperature fluctuations and mechanical vibrations) are shown magnified. This plot is used to determine the root-mean-squared (RMS) current noise, which is determined to be 0.401 μA.

The noise-limited resolution is a key performance metric for airflow sensors, and it reflects the minimum amount of airflow that the sensor can detect above the background noise. In practical terms, the noise-limited resolution indicates the smallest change in airflow that the sensor can measure accurately. The sensor’s response to airflow is compared to its background RMS noise level to determine the noise-limited resolution. The noise-limited resolution for a signal-to-noise ratio (SNR) of 3 is calculated as: 3 × RMS noise/slope (or sensitivity) of the sensor’s response curve. The RMS noise estimated for the 16 sccm flow rate from the inset of Figure 5d is ~0.401 × 10^−6^ A. The sensitivity is determined from the slope of the sensor’s response curve in Figure 5c as 0.307 × 10^−6^ A/sccm. This yields a noise-limited resolution of 3.93 sccm for SNR = 3. Thus, one should be able to resolve flow rates differing by ~4 sccm, which was indeed observed in our measurements (see Figure 5d). For the larger range of flow investigated, i.e., from 100 to 2000 sccm flow rate, the sensitivity and the rms noise were found in Figure 4b,c as 8.9 × 10^−9^ A/sccm and 13.61 × 10^−9^ A, respectively. This yields a volume flow resolution of 4.59 sccm for SNR = 3, which is comparable to the flow resolution mentioned above. Often, the detection resolution for flow velocity is used to characterize a sensor. The flow velocity measurement resolution for the sensor can be determined from the flow rate (in cm/s) resolution simply by using the formula: (flow rate in sccm/(60 × area)), (0.3167 cm^2^ for the high flow range) of the tube. The noise-limited flow velocity resolution is then calculated as 0.241 cm/s for SNR = 3 over the range of 5–105 cm/s. This, to our knowledge, is one of the best air velocity resolutions reported so far in the literature (see Table 1) [38,39,40,41]. The average power consumed by the sensor can be calculated from the applied voltage of 10 V, and the corresponding current varies from 400 to 500 μA, which yields a power consumption of 4–5 mW for our sensor, which is also one of the best reported for these sensors [11,42].

**Figure 6:**
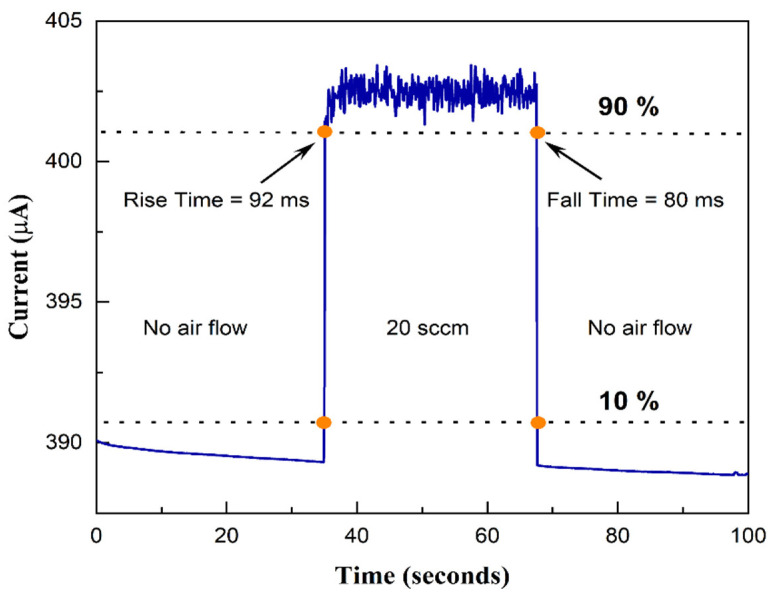
Microcantilever sensor response for 20 sccm airflow. Response time (τ_rise_ = τ_90%_ − τ_10%_), where τ_100%_ is the final value in response to 20 sccm airflow), is calculated to be 92 ms, and recovery time (τ_fall_ = τ_10%_ − τ_90%_) is calculated to be 80 ms seconds.

**Figure 7:**
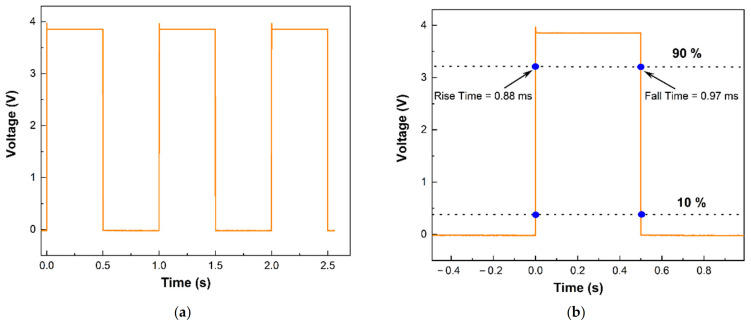
(**a**) Sensor response plotted as a function of time along with the applied voltage pulses; (**b**) a magnified response pulse used for calculating the response and recovery times, which turned out to be <1 ms.

Another critical parameter to characterize a sensor is its response time, which is defined as the time required to reach from 10% to 90% of its final response. To determine the response time, we plotted the response of the sensor for 20 sccm air flow (from Figure 5b) in Figure 6. We find that as the 20 sccm of airflow was introduced, the sensor took 92 ms (τ_rise_) to reach from 10% to 90% of its final response, and it took 80 ms (τ_fall_) to reach from 10% to 90% of its final recovery. The response time, however, includes several factors, including the response time of the MFCs, the flow delay in the tube and in the chamber, and of course, the response time of the sensor itself. The response of the sensor itself will be typically limited by the thermal time constant of the microcantilever, which can be as low as tens of µs for heated microcantilever tips [43,44]. To verify that the thermal time constant is indeed much lower than the response time we observed in Figure 6, we applied voltage pulses to the sensor (0 to 10 V, with 50% duty cycle and 1 Hz frequency) and plotted the sensor response as a function of time in Figure 7a. Note that the sensor response is the voltage measured across a resistor (1 kΩ) in series with the sensor, which is proportional to the current flow. This was performed to measure the current pulse with high temporal resolution using a digital storage oscilloscope. Figure 7b shows the magnified image of a sensor response. The response (rise) and recovery (fall) times extracted from the pulse are 0.88 and 0.97 ms, respectively, which are indicative of the thermal response time of the sensor. The intrinsic sensor response time will also be similar to these times and can be expected to be a few ms. This is also in agreement with the typical time constants of thermal flow sensors reported in the literature [45,46]. Comparing the overall performance metric of our flow sensor to that of other flow and velocity sensors reported so far, we find that it performs very well in terms of low power consumption and flow velocity measurement, as well as intrinsic response time. In terms of flow volume measurement, although there are other specialized sensors that have yielded lower flow rate measurement capabilities even below 1 sccm [47,48], their geometry is typically more complicated [49] with an integrated heater coil and two thermistors on two sides. The performance of our sensor, with its very simple geometry, can still be desirable in many applications due to its very small active area and simple two-terminal measurement.

We would like to mention here that, in terms of the choice of semiconductor materials to realize the heated apex flow sensor, Si can also be a good choice. However, since the sensor is based on the reduction in the differential temperature (compared to ambient) of the cantilever apex and the ambient is at a higher temperature, i.e., 200 °C, and the apex needs to be at 300 °C, Si-based devices may begin to face operational issues. Apart from this, there are several other reasons why the AlGaN/GaN heterostructure-based sensor was investigated for flow sensing, which are discussed below. First, due to the localized sheet carrier density (2DEG) present at the interface of AlGaN/GaN, (aided by the special triangular geometry of the cantilever), it is much more efficient to concentrate heat at the apex of the cantilever as opposed to a Si-based sensor where the carriers are in 3D. Second, the hottest point being only ~20 nm below the surface helps with sensor sensitivity by enhancing interaction with the oncoming air flow. These reasons resulted in lower power consumption for these sensors and increased sensitivity, as noted above. Finally, these III-Nitride sensors also offer the possibility of integration with other sensors and high-performance electronics, like other MEMS sensors and high-performance transistors, resulting in the development of an integrated system with multifunctional sensing and processing capabilities.

## 4. Conclusions

In conclusion, we have investigated a heated apex triangular microcantilever for airflow sensing using a very simple two-terminal sensor configuration. Thermal microscope images confirm that the apex region of the microcantilever reaches significantly higher temperatures than other parts. The sensor response was found to vary linearly with airflow rate when tested over a range of airflow varying from 16 to 2000 sccm. The noise-limited flow volume measurement yielded 4 sccm resolution for an SNR of 3. For the same SNR, the velocity resolution was found to be 0.241 cm/s, which is one of the best reported so far for thermal sensors. The power consumption of the sensor was also one of the lowest reported at ~5 mW, while the intrinsic response time was estimated to be on the order of a few ms. Overall, the microcantilever sensor, with its simple geometry and measurement configurations, was found to exhibit attractive performance metrics that can be useful for a variety of sensing applications.

## Figures and Tables

**Table 1:** Comparison of sensors for detecting airflow velocities.

Sensing Material	Detection Range (cm/s)	Flow Velocity Resolution	Response Time	Power Consumption	Ref.
3C-SiC on Glass	0 to 900	9.11 cm/s	2 s	133.50 mW	[38]
Ni resistor	0 to 3000	2.93 cm/s	-	256 mW	[39]
SOI sensing cantilever	200 to 2000	10 cm/s	-	55.20 mW	[40]
Pd reference electrode	66 to 97	2.5 cm/s	-	-	[41]
AlGaN/GaN triangular microcantilever	5 to 105	0.241 cm/s	92 ms (<1 ms intrinsic)	5 mW	This work

## Supplementary Materials

The following supporting information can be downloaded at: https://www.mdpi.com/article/10.3390/s23177465/s1, Figure S1: Triangular microcantilever fabrication process flow; Figure S2: (**a**) Airflow detection from 300 sccm to 1000 sccm using a different microcantilever heater of similar dimensions, (**b**) detection for a varying airflow from 300 sccm to 1000 sccm with a step size of 100 sccm, (**c**) airflow detection with 2000 sccm flow rate. For all the flow measurement experiments the voltage bias was maintained at 10 V.
