# Supplementary material for: Low-Power AlGaN/GaN Triangular Microcantilever for Air Flow Detection
Source: Sensors (Basel). 2023 Aug 28;23(17):7465. doi: 10.3390/s23177465 (PMC10490568; doi:10.3390/s23177465)
Supplement: Supplementary file 1 [file sensors-23-07465-s001.zip › sensors-2503627-supplementary.pdf]

Low power AlGaN/GaN Triangular Microcantilever for Air Flow Detection

Balaadithya Uppalapati[1*], Durga Gajula[2], Manav Bava[1], Lavanya Muthusamy[1], and Goutam Koley[1]

[1]*Holcombe Department of Electrical and Computer Engineering, Clemson University, Clemson, SC 29634, USA*
[2]*School of Electrical and Computer Engineering, Georgia Institute of Technology, Atlanta, GA 30332, USA*
[*]*Correspondence should be addressed to: buppala@clemson.edu*

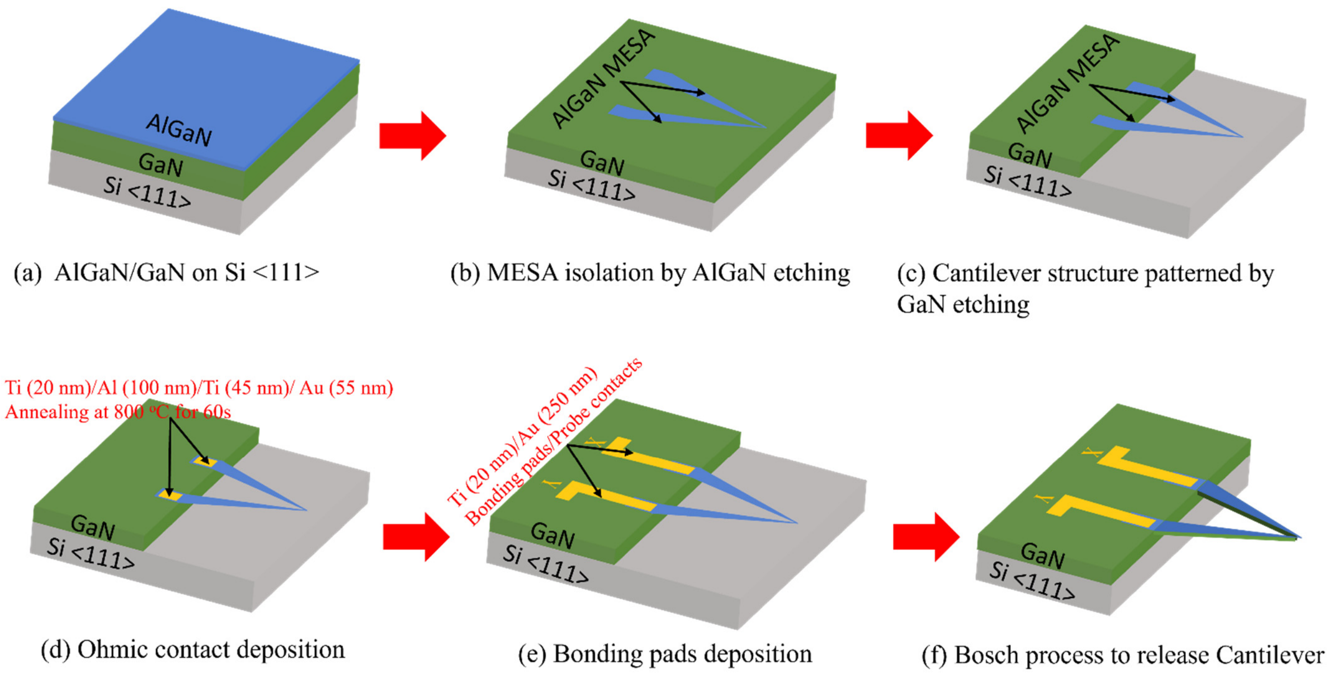

Figure S1. Triangular microcantilever fabrication process flow

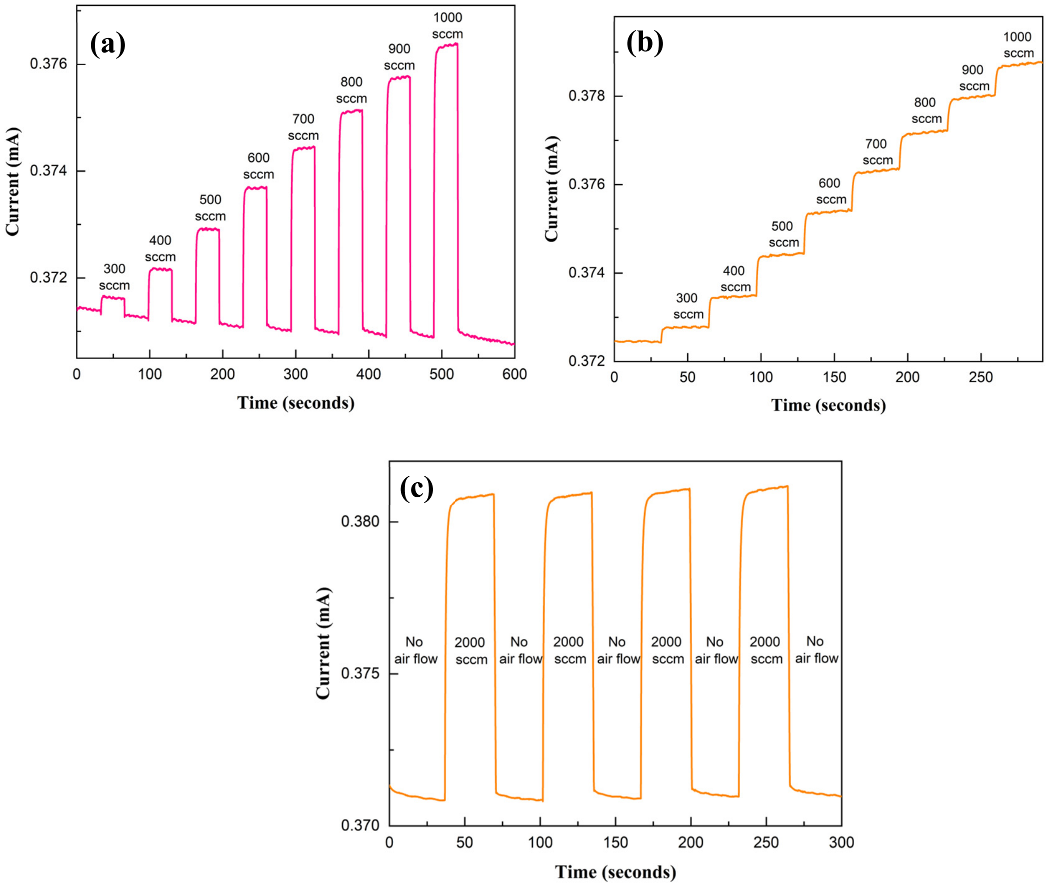

Figure S2. (a) Airflow detection from 300 sccm to 1000 sccm using a different microcantilever heater of similar dimensions, **(b)** detection for a varying airflow from 300 sccm to 1000 sccm with a step size of 100 sccm, **(c)** airflow detection with 2000 sccm flow rate. For all the flow measurement experiments the voltage bias was maintained at 10 V.
